# B7-H3 (CD276) and CD47 Expression Are Associated with Immune Evasion and Survival Outcomes in Pediatric Medulloblastoma

**DOI:** 10.3390/diagnostics16101419

**Published:** 2026-05-07

**Authors:** Eray Abat, Ezgi Gizem Abat, Yusuf Emrullahoğlu, Ramazan Oğuz Yüceer, Süleyman Mazıcı, Nimetullah Alper Durmuş, Ali Şahin, Halil Ulutabanca, Şükrü Oral, Alper Özcan, Özlem Canöz, Ahmet Küçük

**Affiliations:** 1 Department of Neurosurgery, Develi Dr. Ekrem Karakaya State Hospital, Kayseri 38400, Turkey; eray_abat@hotmail.com; 2 Department of Pathology, Kayseri City Hospital, Kayseri 38000, Turkey; ezgi2372@hotmail.com; 3 Department of Neurosurgery, Faculty of Medicine, Erciyes University, Kayseri 38030, Turkey; dr.yusufemrullahoglu@gmail.com (Y.E.); nimetullahalper@hotmail.com (N.A.D.); dralishn@gmail.com (A.Ş.); ulutabanca@erciyes.edu.tr (H.U.); ahmetkucuk@erciyes.edu.tr (A.K.); 4 Department of Pathology, Faculty of Medicine, Cumhuriyet University, Sivas 58140, Turkey; r.yuceer66@hotmail.com; 5 Department of Pediatric Neonatology, Faculty of Medicine, Erciyes University, Kayseri 38030, Turkey; s.mazici@hotmail.com; 6 Department of Pediatric Hematology and Oncology, Faculty of Medicine, Erciyes University, Kayseri 38030, Turkey; dralperozcan@hotmail.com; 7 Department of Pathology, Faculty of Medicine, Erciyes University, Kayseri 38030, Turkey; ocanoz@gmail.com

**Keywords:** medulloblastoma, B7-H3 (CD276), CD47, immune checkpoint, immunohistochemistry, overall survival, disease-free survival, pediatric brain tumor

## Abstract

**Background:** Medulloblastoma is the most common malignant pediatric central nervous system tumor and exhibits marked molecular and clinical heterogeneity. Immune evasion pathways may contribute to tumor aggressiveness and represent potential prognostic and therapeutic targets. We investigated the clinicopathological and prognostic relevance of the immune checkpoint molecules B7-H3 (CD276) and CD47 in medulloblastoma. **Methods:** We screened 77 patients treated between April 2015 and December 2025; in total, 32 patients with pathologically confirmed medulloblastoma and complete data were included. Tumor B7-H3 and CD47 expression was assessed using immunohistochemistry and an immunoreactivity score (IRS); patients were categorized as having negative/low (IRS < 4) or high (IRS ≥ 4) expression. We analyzed the associations with clinicopathological and molecular features. Overall survival (OS) and disease-free survival (DFS) were evaluated using Kaplan–Meier and log-rank tests. Prognostic factors were examined using univariate and multivariate Cox regression. **Results:** High B7-H3 expression was associated with shorter OS (median 46 vs. 85 months; *p* = 0.024) and markedly reduced DFS (median 21 vs. 102 months; *p* < 0.001). High CD47 expression was also associated with shorter OS (median 56 vs. 102 months; *p* = 0.025), whereas DFS did not significantly differ by CD47 status (*p* = 0.200). B7-H3 and CD47 expression levels were not correlated (Spearman’s rho = 0.071; *p* = 0.699). In the univariate analysis, high B7-H3 expression predicted mortality (HR = 25.79; *p* = 0.002) and recurrence risk (HR = 136.23; *p* = 0.045), and high CD47 expression predicted mortality (HR = 4.82; *p* = 0.042). In the multivariate analysis, high B7-H3 expression remained an independent predictor of poor OS (HR = 31.01; *p* = 0.004), whereas radiotherapy independently reduced the recurrence risk (HR = 0.197; *p* = 0.024). **Conclusions:** B7-H3 is a strong independent adverse prognostic biomarker for OS and is associated with profoundly shorter DFS in medulloblastoma, supporting its relevance as a candidate target for immune-directed strategies.

## 1. Introduction

Medulloblastoma is the most common malignant central nervous system tumor of childhood and remains a major clinical challenge owing to its heterogeneous biological behavior and marked molecular diversity [1]. The current World Health Organization classification delineates four principal molecular subgroups—WNT, SHH, Group 3, and Group 4—each associated with distinct prognostic patterns and therapeutic implications [2]. However, this subtype-based framework does not fully capture the complexity of tumor micromorphology, the immune microenvironment, or the contribution of immune-regulatory molecules to tumor progression [3]. In clinically aggressive entities, such as SHH-p53 mutant and Group 3 medulloblastomas, conventional clinical and pathological indicators frequently fail to predict biological behavior with sufficient accuracy, underscoring the need for novel prognostic biomarkers [4].

In recent years, the role of the tumor immune microenvironment in the pathogenesis and progression of medulloblastoma has gained increasing attention, and molecules involved in immune evasion pathways have emerged as potential therapeutic targets [5]. In this context, B7-H3 and CD47 have drawn particular interest as two key immune-regulatory molecules that operate at distinct checkpoints and are capable of reshaping interactions between tumor cells and the host immune system [6,7].

B7-H3 is an immune-regulatory transmembrane glycoprotein of the B7 family that is markedly overexpressed in tumor tissues compared to normal physiological counterparts. Its immunosuppressive activity is mediated through the inhibition of T-cell proliferation, reduction in cytotoxic T-cell function, and promotion of immune escape within the tumor microenvironment [8,9]. In addition to its immunological effects, B7-H3 contributes to several fundamental processes in cancer biology, including tumor cell invasion, angiogenesis, epithelial–mesenchymal transition, and chemoresistance [10,11]. In medulloblastoma, the observation that B7-H3 expression is higher in more aggressive molecular subtypes suggests that this molecule may have prognostic relevance, as well as potential value as a therapeutic target [12,13].

CD47 represents a central innate immune checkpoint that delivers the “do not eat me” signal, thereby enabling tumor cells to evade macrophage-mediated clearance. By binding to the SIRP-alpha receptor on macrophages, CD47 suppresses phagocytosis and protects tumor cells from antitumor immune responses [14,15]. High CD47 expression in solid malignancies has been associated with increased invasiveness, metastatic potential, stem-like cellular features, and reduced survival [16,17,18]. Although CD47-mediated immune evasion mechanisms have been suggested to play a role in pediatric medulloblastoma, its subtype-specific relevance, particularly within Group 3 tumors, remains insufficiently characterized and warrants further investigation [3,7].

Although these two molecules operate at distinct levels of immune regulation, they share a common function in helping tumor cells avoid recognition and elimination by the immune system. B7-H3 primarily suppresses T cell-mediated adaptive immunity, whereas CD47 inhibits macrophage-driven clearance as part of the innate immune response [8,14]. Through the combined restraint of both adaptive and innate immune pathways, the tumor can establish a dual-layered mechanism of immune escape. Accordingly, the simultaneous evaluation of B7-H3 and CD47 provides a valuable opportunity to delineate the multifaceted nature of immune evasion in medulloblastoma.

In addition, studies that evaluate both molecules within the same medulloblastoma cohort, particularly those incorporating comparative and survival-focused analyses, are scarce in the literature. Comprehensive clinicopathological investigations with long-term overall survival (OS) and disease-free survival (DFS) outcomes are especially limited in pediatric populations. Addressing this gap by examining B7-H3 and CD47 in parallel may clarify not only their prognostic relevance but also inform the development of future immunotherapeutic strategies.

In this study, we analyzed the expression of B7-H3 and CD47 in medulloblastoma cases and examined their associations with clinical, histopathological, and molecular subgroups as well as their impact on survival outcomes. Our findings may contribute to the development of new prognostic approaches that more accurately reflect the immunological landscape of medulloblastoma.

## 2. Materials and Methods

### 2.1. Patient Selection, Data Collection, and Clinical Follow-Up

In this retrospective study, patients with a pathologically confirmed diagnosis of medulloblastoma according to the 2021 World Health Organization (WHO) Classification of Central Nervous System Tumors were evaluated [19]. A total of 77 patients treated at the Erciyes University Faculty of Medicine Research and Application Hospital between April 2015 and December 2025 constituted the initial cohort.

The predefined exclusion criteria were as follows: absence of surgical resection (*n* = 10), insufficient tumor tissue for immunohistochemical analysis (*n* = 8), use of immunosuppressive therapy due to chronic inflammatory disease (*n* = 6), and incomplete or unavailable clinical follow-up data (*n* = 11). After applying these criteria, 32 patients with complete clinical, radiological, and laboratory data were included in the final study cohort.

The collected data included demographic and clinical characteristics, such as age, sex, Eastern Cooperative Oncology Group performance status (ECOG PS), and comorbidities, as well as tumor-related variables, including tumor lateralization, lobe of origin, focality, and tumor size. In addition, surgical approach, Ki-67 proliferation index, p53 mutation status, alpha-thalassemia/mental retardation syndrome X-linked (ATRX) loss, isocitrate dehydrogenase (IDH) mutation status, administered adjuvant therapies, treatment response, disease progression, subsequent treatment strategies, and survival outcomes were recorded.

Molecular subgroup classification (WNT, SHH, Group 3, and Group 4) was determined based on immunohistochemical (IHC) staining patterns. Representative biomarkers were applied on formalin-fixed, paraffin-embedded tissue sections using an automated staining platform. The classification was performed according to established IHC-based surrogate algorithms reported in the literature [20]. Cases were categorized into molecular subgroups by integrating the expression profiles of the applied markers.

All patients were treated according to multidisciplinary decisions based on current pediatric medulloblastoma treatment principles. Initial treatment consisted of maximal safe surgical resection, followed by risk-adapted adjuvant radiotherapy and/or chemotherapy when clinically indicated. Patients were regularly monitored throughout the treatment and follow-up periods.

Clinical responses were assessed according to the Revised Response Evaluation Criteria in Solid Tumors (RECIST), version 1.1. Disease-free survival (DFS) was defined as the time from the date of pathological diagnosis to disease progression, death, or the last follow-up visit. Overall survival (OS) was calculated as the time from the date of pathological diagnosis to death from any cause or the last follow-up.

### 2.2. Outcomes of the Study

The study endpoints included overall survival (OS) and disease-free survival (DFS). OS was defined as the time from the date of pathological diagnosis to death from any cause or the last follow-up visit. DFS was defined as the time from the date of pathological diagnosis to disease progression, recurrence, death, or the last follow-up visit. Survival outcomes were assessed using clinical follow-up records and radiological evaluations.

### 2.3. Immunohistochemical Evaluation of B7-H3 and CD47 Expression in Tumor Tissues

Hematoxylin and eosin (H&E)-stained tumor sections from patients with medulloblastoma were re-evaluated, and paraffin blocks containing sufficient tumor tissue without necrotic areas were selected. Sections with a thickness of 4 µm were cut from these blocks and mounted on adhesive-coated slides.

For immunohistochemical staining, B7-H3 (rabbit monoclonal antibody, clone EPR20115, 1:1000 dilution; Abcam, Cambridge, UK) and CD47 (rabbit monoclonal antibody, clone EPR21794, 1:1000 dilution; Abcam, Cambridge, UK) primary antibodies were used. Tonsil tissue served as the positive control for both B7-H3 and CD47, and was processed simultaneously with the tumor samples.

All immunohistochemical staining procedures were performed using a fully automated staining system (Roche Ventana Benchmark Ultra, Basel, Switzerland). The stained slides were independently evaluated by experienced neuropathologists who were blinded to the clinical data. Expression levels were calculated using the immunoreactivity score (IRS), a validated semi-quantitative method for immunohistochemical assessment.

**Figure 1:**
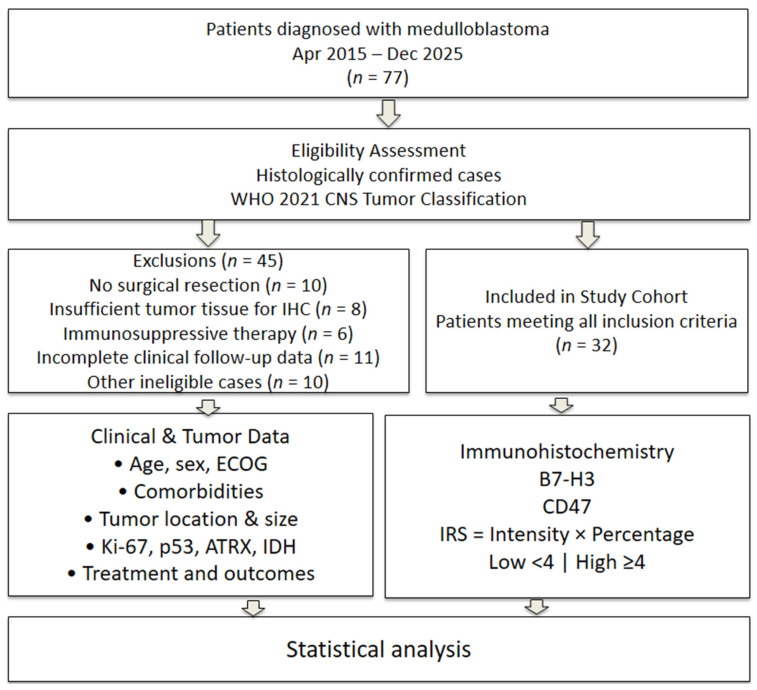
Flow diagram illustrating the patient selection process.

Membranous and/or cytoplasmic staining was considered positive for both B7-H3 and CD47. Staining intensity was graded as 0 (no staining), 1 (weak), 2 (moderate), or 3 (strong), while the proportion of positive tumor cells was scored as 0 (0%), 1 (1–19%), 2 (20–50%), or 3 (>50%). The final IRS was obtained by multiplying staining intensity by the proportion score. Based on this value, samples were categorized as having low (IRS < 4) or high (IRS ≥ 4) expression. This approach is consistent with the commonly used semi-quantitative immunohistochemical evaluation methods reported in the literature [14] (Figure 1).

### 2.4. Statistical Analysis

All statistical analyses were conducted using IBM SPSS Statistics for Windows, version 27 (IBM Corp., Armonk, NY, USA), and R statistical software (version 4.x; R Foundation for Statistical Computing, Vienna, Austria). Normality of continuous variables was assessed using the Kolmogorov–Smirnov test. Variables with a normal distribution are presented as mean ± standard deviation, whereas non-normally distributed variables are expressed as medians with ranges. Associations between categorical variables, including B7-H3 and CD47 expression and clinicopathological parameters, were evaluated using Pearson’s χ^2^ test or Fisher’s exact test when appropriate. Spearman’s rank correlation analysis was performed to examine non-parametric relationships between variables.

OS and disease-free survival (DFS) were estimated using the Kaplan–Meier method, and differences between survival curves were compared using the log-rank test. Kaplan–Meier survival plots and corresponding risk tables were generated using the “survival” and “survminer” packages in R. Variables included in the multivariate Cox regression model were selected based on their clinical relevance and biological plausibility. Multicollinearity among variables was assessed using the variance inflation factor (VIF), and no variable exceeded the predefined threshold (VIF < 4.0). These approaches were used to reduce the risk of model instability associated with the limited sample size. Hazard ratios (HRs) with corresponding 95% confidence intervals (CIs) were calculated. Statistical significance was set at *p* < 0.05.

## 3. Results

### 3.1. Patient and Tumor Characteristics

The median age of the patients included in the study was 9 years (interquartile range [IQR], 1–18 years). Approximately 59.4% of the cases were ≤9 years of age, and a clear male predominance was observed (68.8%).

Histologically, the classic subtype was the most common (65.6%), followed by the desmoplastic–nodular variant (21.9%). At the molecular level, the MB-SHH p53-mutant subgroup was the most commonly identified (34.4%), followed by MB-Group 3 (28.1%) and MB-WNT (18.8%). Overall, p53 mutations were detected in 53.1% of the cases.

A Ki-67 proliferation index greater than 10% was observed in 56.3% of patients, suggesting increased biological aggressiveness.

Regarding tumor localization, lesions were most frequently located in the cerebellar vermis (37.5%), followed by hemispheric locations (31.3%).

Most cases (93.8%) showed no detectable genetic syndrome; however, Li-Fraumeni syndrome (3.1%) and neurofibromatosis (3.1%) were rarely identified.

The tumor size ranged between 3 and 6 cm (65.6%). Metastatic disease was present in 56.3% of patients, predominantly among those with advanced pathological stages (pT3a–pT3b and pT4).

Gross total resection (GTR) was achieved in 50% of the cases. Radiotherapy was administered to 78.1% of the patients, whereas 68.8% received chemotherapy.

Within the MB-WNT subgroup, approximately two-thirds of the patients were older than 9 years (66.7%), whereas an earlier onset at ≤9 years of age was observed more frequently in the MB-G3 and MB-G4 subtypes (77.8% and 100.0%, respectively).

Regarding sex distribution, all cases in the MB-SHH p53-wild-type subgroup were men (100.0%).

From a histopathological perspective, the classic subtype was the predominant histological pattern. The prevalence of classic histology was 83.3% in MB-WNT and 77.8% in MB-G3, whereas the desmoplastic–nodular variant was particularly prominent in the MB-SHH p53-mutant subgroup (45.5%).

The p53 status was completely concordant with the molecular classification. All cases in the MB-SHH p53-mutant subgroup showed mutant p53 expression (100.0%), whereas all MB-WNT cases exhibited wild-type p53 (100.0%).

An elevated Ki-67 proliferation index (≥10%) was more frequently observed in biologically aggressive subtypes, particularly in the MB-G3 (66.7%) and MB-SHH p53-mutant (54.5%) groups.

In terms of metastatic disease, the MB-G3 subgroup showed the highest rate of metastatic spread (88.9%).

In terms of surgical management, gross total resection (GTR) was most commonly achieved in the MB-WNT (83.3%) and MB-SHH p53-mutant (63.6%) subtypes.

Radiotherapy was administered to all patients in the MB-WNT, MB-G4, and MB-SHH p53-mutant subgroups (100%) (Table 1).

### 3.2. B7-H3 and CD47 Expression and Clinicopathological Characteristics

Cases of medulloblastoma with low (*n* = 11) and high (*n* = 21) B7-H3 expression were compared in terms of clinical and biological parameters.

In the high B7-H3 expression group, the proportion of patients aged >9 years (42.9%) was higher than that in the low-expression group; however, this difference was not significant (*p* = 0.513). Male sex predominance was observed in both groups, accounting for 71.4% of patients in the high-expression group.

Regarding histological distribution, classic medulloblastoma was more frequently observed in cases with high B7-H3 expression (71.4% vs. 54.5%), whereas the desmoplastic/nodular subtype was slightly more common in the low-expression group (27.3%).

Cases with a Ki-67 proliferation index ≥ 10% were markedly more frequent in the high B7-H3 expression group (66.7% vs. 36.4%), suggesting a possible association between increased B7-H3 expression and enhanced proliferative activity.

N-Myc amplification was also more frequently observed in the high-expression group (23.8% vs. 9.1%). Similarly, advanced pathological stages (pT3a–pT3b) were more common among cases with high B7-H3 expression (47.6%).

With respect to treatment approaches, all patients with low B7-H3 expression received radiotherapy (100%), whereas the rate of radiotherapy in the high-expression group was 66.7% (*p* = 0.025).

In terms of molecular subgroup distribution, MB-G3 and MB-SHH p53-mutant cases were more prevalent in the high B7-H3 expression group.

Cases of medulloblastoma with negative/low (*n* = 13) and high (*n* = 19) CD47 expression were compared according to clinicopathological parameters.

In patients with high CD47 expression, the proportion of cases aged ≤9 years was predominant (68.4%), whereas in the negative/low expression group, the proportion of patients aged >9 years was relatively higher (53.8%). Male predominance was more evident in the high CD47 expression group (78.9% vs. 53.8%).

Among cases with high CD47 expression, the classic subtype was the most common histological pattern (68.4%).

A Ki-67 proliferation index ≥ 10% was more frequently observed in the high CD47 expression group (68.4% vs. 38.5%), suggesting a possible association between CD47 positivity and increased proliferative activity.

With respect to metastatic disease, metastasis was more common in the low CD47 expression group (69.2%), whereas it was observed in 47.4% of patients with high CD47 expression.

With respect to surgical treatment, the rate of gross total resection (GTR) was significantly higher in the high CD47 expression group (63.2%) than in the negative/low expression group (30.8%) (*p* = 0.032).

In the molecular subgroup distribution, the proportion of MB-SHH p53-mutant cases was similar between the two groups (low CD47: 38.5%; high CD47: 31.6%). The MB-G3 subtype was observed at moderate frequencies in both groups (30.8% and 26.3%, respectively).

**Figure 2:**
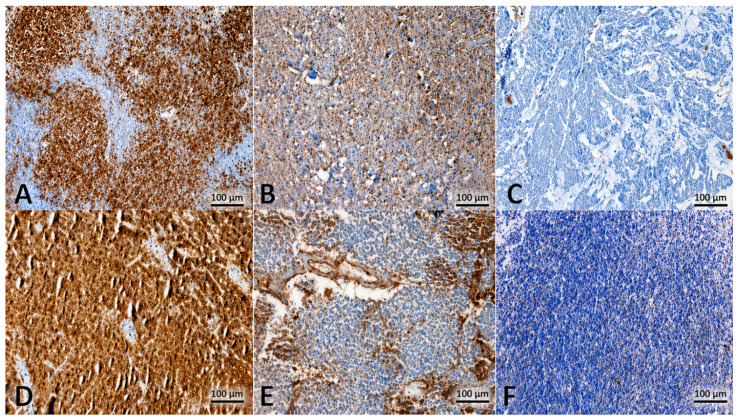
Immunohistochemical expression patterns of B7-H3 and CD47 in medulloblastoma. (**A**) High B7-H3 expression showing strong membranous and cytoplasmic immunoreactivity in tumor cells. (**B**) Low B7-H3 expression characterized by weak and focal staining. (**C**) Negative B7-H3 staining demonstrating absence of immunoreactivity in tumor cells. (**D**) High CD47 expression with strong membranous staining in tumor cells. (**E**) Low CD47 expression showing weak and partial immunoreactivity. (**F**) Negative CD47 staining with no detectable immunoreactivity. Scale bar: 100 µm (DAB (3,3′-diaminobenzidine)× 200).

Spearman’s correlation analysis demonstrated no significant association between B7-H3 and CD47 expression levels (rho = 0.071; *p* = 0.699). The low and weakly positive correlation coefficient suggests that the expression patterns of these two immune checkpoint molecules in tumor tissue likely occur independently of each other (Table 2 and Table 3, Figure 2).

### 3.3. Survival Analysis

During a median follow-up of 74.6 months, 18 patients (56.3%) died. The median overall survival (OS) was 68 months (95% CI: 48.9–87.0).

Patients with low B7-H3 expression had a median OS of 85 months (95% CI: 46.3–123.7), whereas those with high B7-H3 expression showed a significantly shorter median OS of 46 months (95% CI: 14.8–77.2) (*p* = 0.024).

**Figure 3:**
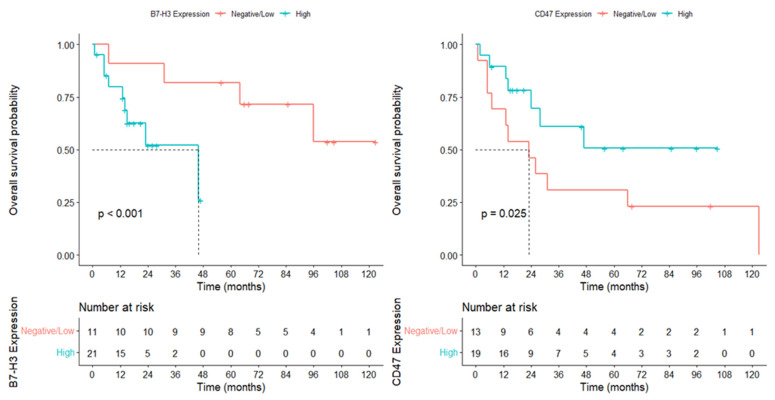
Kaplan–Meier overall survival (OS) curves according to B7-H3 and CD47 expression in medulloblastoma.

Similarly, patients with low CD47 expression had a median OS of 102 months (95% CI: 47.6–156.4), whereas the high CD47 expression group demonstrated a significantly shorter median OS of 56 months (95% CI: 4.8–107.2) (*p* = 0.025) (Figure 3).

During the follow-up, tumor recurrence occurred in 19 patients (59.4%). The median disease-free survival was 47 months (95% confidence interval [CI], 6.9–87.1).

**Figure 4:**
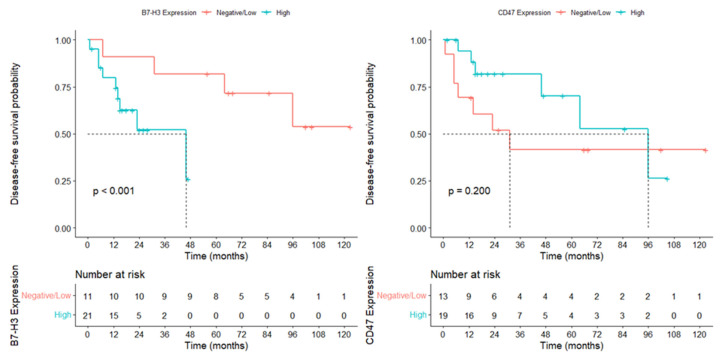
Kaplan–Meier disease-free survival (DFS) curves according to B7-H3 and CD47 expression in medulloblastoma.

Patients with low B7-H3 expression had a median DFS of 102 months (95% CI: 61.2–142.9), whereas in patients with high B7-H3 expression, this duration decreased dramatically to 21 months (95% CI: 12.6–29.4) (*p* < 0.001). In cases with low CD47 expression, the median DFS was 68 months (95% CI: 63.7–72.3), while the high CD47 expression group had a median DFS of 28 months (95% CI: 0–59.0); however, this difference did not reach statistical significance (*p* = 0.200) (Figure 4).

Univariate OS analysis identified high B7-H3 expression as the strongest predictor of mortality (HR = 25.79, *p* = 0.002). High CD47 expression was also associated with poorer survival (HR = 4.82, *p* = 0.042). The presence of genetic syndromes negatively affected survival, whereas radiotherapy (HR = 0.087) and chemotherapy (HR = 0.266) significantly reduced mortality risk. In the DFS analysis, B7-H3 overexpression emerged as the most powerful adverse prognostic factor (HR = 136.23, *p* = 0.045). Radiotherapy significantly decreased the risk of recurrence, whereas CD47 expression showed only a borderline association with DFS (Table 4).

In the multivariate Cox regression analysis, high B7-H3 expression remained the strongest independent predictor of poor OS (HR = 31.01, *p* = 0.004). CD47 expression, radiotherapy, chemotherapy, and genetic anomalies were not independently associated with OS. Radiotherapy emerged as the only independent protective factor for DFS, significantly reducing the risk of recurrence (HR = 0.197, *p* = 0.024). Although B7-H3 expression showed strong prognostic significance in the univariate DFS analysis, it did not retain statistical significance in the multivariate model, suggesting a clinically relevant but non-independent effect (Table 5).

## 4. Discussion

In this study, we evaluated the clinical relevance of immune checkpoint molecules B7-H3 and CD47 in pediatric medulloblastoma. Both markers were found to be expressed at notable levels within the tumor tissue, and high B7-H3 expression was associated with shorter overall and disease-free survival. High CD47 expression was associated with poorer overall survival in the univariate analysis, whereas no clear relationship was observed with metastasis, disease-free survival, or established clinical risk groups. In contrast to B7-H3, the impact of CD47 appeared to be more limited and confined to overall survival. These findings suggest that B7-H3 and CD47 may influence tumor behavior through different immune-related mechanisms rather than acting within a single unified pathway of immune escape.

In our cohort, B7-H3 expression was markedly elevated in medulloblastoma tissue, and higher expression levels were associated with poorer clinical outcomes. The relationship between increased B7-H3 expression, reduced survival, and a tendency toward metastatic disease is consistent with prior studies highlighting the prognostic relevance of B7-H3 in medulloblastoma. Fontão et al. similarly reported that elevated B7-H3 expression correlated with metastasis and unfavorable prognosis [21]. This concordance supports the notion that B7-H3 may serve as a meaningful biomarker of clinical behavior in medulloblastoma.

The role of B7-H3 in medulloblastoma is supported by both clinical and molecular evidence of tumor aggressiveness. In a study by Marques et al., elevated B7-H3 expression in medulloblastoma was linked to more aggressive molecular phenotypes, including unfavorable prognostic profiles and increased invasive behavior within immune checkpoint gene signatures [22]. These molecular findings align with our results, in which higher B7-H3 expression corresponded with metastatic tendencies and poorer survival, providing a biological context for the clinical associations observed in our cohort.

Beyond medulloblastoma, B7-H3 has been implicated in immune evasion and tumor progression across a range of primary brain tumors. Recent studies have highlighted the multifaceted role of B7-H3, including its involvement in T-cell inhibition, establishment of an immunosuppressive microenvironment, tumor angiogenesis, stromal remodeling, glioma progression and glioma cell invasion and migration [23,24,25]. The consistently high expression of B7-H3 across brain malignancies suggests that it operates as a central node in immune-escape pathways and contributes to the attenuation of antitumor immune responses. This broader mechanistic framework aligns with our findings, in which elevated B7-H3 expression corresponded with a more aggressive clinical phenotype, providing additional biological context for the associations observed in our cohort.

Similarly, Yüceer et al. (2025) investigated B7-H3 expression in patients with IDH-wild-type glioblastoma and reported that elevated B7-H3 levels were independently associated with overall survival [26]. Their findings showed that increased B7-H3 expression was associated with poorer clinical outcomes and suggested that B7-H3 may serve as a promising prognostic biomarker in glioblastoma. These observations are consistent with the findings of the present study [26].

In our cohort, CD47 expression was markedly elevated in medulloblastoma tissues and appeared to influence survival outcomes. In the univariate analysis, high CD47 levels were associated with significantly poorer overall survival (HR = 4.816; *p* = 0.042), suggesting that CD47 may contribute to a more aggressive tumor phenotype. However, no statistically significant associations were observed between CD47 expression and metastasis or disease-free survival. Given the limited number of clinical cohort studies examining CD47 expression specifically in medulloblastoma, these findings represent one of the earliest clinical indications of its potential prognostic relevance. Considering CD47’s established role in suppressing macrophage-mediated antitumor immunity through the “do not eat me” signal, our results are consistent with this biological mechanism and support its involvement in medulloblastoma progression [14].

Extensive studies of various solid tumors have shown that CD47 influences tumor biology not only through immune evasion mechanisms but also by directly enhancing invasion, migration, and metastatic behavior. Feng et al. reported that CD47 overexpression reinforces an aggressive tumor phenotype by modulating extracellular matrix interactions, integrin-mediated signaling, and stromal remodeling [27]. Consistent with these findings, elevated CD47 levels have been linked to poor survival outcomes in multiple cancer types [28]. Although clinical data on medulloblastoma remain limited, this broader biological framework provides a compelling rationale for CD47 to contribute meaningfully to tumor progression in this disease.

One of the best-characterized functions of CD47 in tumor biology is its role in sustaining the ‘do not eat me’ signal that modulates macrophage-mediated immune responses. By engaging the SIRPα receptor on macrophages, CD47 triggers an inhibitory signaling cascade that suppresses phagocytosis, thereby enabling tumor cells to evade innate immune clearance. Liu et al. highlighted this axis as a key contributor to immune suppression within the tumor microenvironment and demonstrated that CD47 overexpression is associated with immune evasion phenotypes across multiple malignancies [29]. This mechanistic framework aligns with our findings, in which elevated CD47 expression was correlated with poorer overall survival, supporting the notion that CD47 may influence disease progression in medulloblastoma through its immunoregulatory effects.

The relevance of CD47 in pediatric brain tumors becomes even more pronounced when considering preclinical evidence. In the study titled “Disrupting the CD47–SIRPα anti-phagocytic axis by a humanized anti-CD47 antibody,” the humanized antibody (Hu5F9-G4) demonstrated potent antitumor activity in malignant pediatric brain tumor models, enhancing macrophage-mediated phagocytosis, and resulting in significant tumor reduction and improved survival [30]. In addition to its prognostic significance, CD47 appears to have potential therapeutic relevance as an immune checkpoint in medulloblastoma. The association between high CD47 expression and poor survival supports further investigation of CD47-targeted therapies.

Interest in immune checkpoint pathways in medulloblastoma has increased in recent years; however, most available studies have focused on individual biomarkers. Although B7-H3 has been more extensively investigated, clinical evidence regarding CD47 remains limited and is predominantly based on preclinical observations. Importantly, these two molecules have not been evaluated together within the same clinical cohort. In this context, our study examined B7-H3 and CD47 in parallel to better characterize immune escape mechanisms and to clarify the clinical relevance of CD47.

Immune escape in medulloblastoma is likely to involve multiple checkpoint pathways. In our cohort, B7-H3 and CD47 were co-expressed in tumor tissues, suggesting potentially complementary roles. Given that B7-H3 is associated with T-cell regulation and CD47 with macrophage-mediated phagocytosis, these findings may reflect the involvement of both adaptive and innate immune responses. However, as this study is based on immunohistochemical and survival analyses, these observations should be interpreted as associative, and not as direct mechanistic evidence.

A strength of this study is the parallel evaluation of B7-H3 and CD47 within the same clinical cohort, allowing a direct comparison of their clinical associations. The integration of histopathological findings with survival data provides additional context for their potential relevance in disease behavior, while remaining within the descriptive scope of the study.

While our findings suggest a potential association with immune evasion, definitive mechanistic conclusions cannot be drawn from this study alone, and further functional studies are required to validate the roles of B7-H3 and CD47 in the medulloblastoma immune microenvironment.

Several limitations should be taken into account. The small sample size and uneven distribution of molecular subtypes may have influenced the results of the subgroup analyses. A key limitation of this study is the very small sample size in certain molecular subgroups, particularly Group 4. This restricts the statistical power of subgroup analyses and limits the reliability and generalizability of comparisons. The single-center nature of the study may also limit its generalizability. In addition, the absence of functional studies means that the findings are based on immunohistochemical and clinical associations rather than direct mechanistic evidence. In addition, the relatively large hazard ratios and wide confidence intervals observed in some analyses should be interpreted cautiously, as they may reflect the limited sample size and reduced statistical stability of the estimates. Larger multicenter studies with functional validation would help to further clarify these findings.

## 5. Conclusions

In this study, we evaluated the clinical relevance of B7-H3 and CD47 expression in pediatric medulloblastoma and found that these two immune-regulatory molecules may be associated with tumor biology through distinct pathways. Higher B7-H3 expression was associated with poorer overall and disease-free survival, indicating its potential role as a prognostic marker. In contrast, CD47 expression was mainly related to overall survival and did not show clear associations with other clinical parameters. The co-expression of both markers in the same tumor samples suggests a possible association with multiple immune escape mechanisms in medulloblastoma. These findings support a complementary role for B7-H3 and CD47 and highlight their potential relevance in future immune-targeted strategies; further validation in larger and functionally supported studies is warranted.

## Figures and Tables

**Table 1:** Clinicopathological characteristics according to molecular subtype of medulloblastoma.

Variable	Category	MB-WNT *n* (%)	MB-SHH p53 Mutant *n* (%)	MB-SHH p53 Wild-Type *n* (%)	MB-Group 3 *n* (%)	MB-Group 4 *n* (%)	*p*
Age	≤9 years	2 (33.3)	6 (54.5)	3 (60.0)	7 (77.8)	1 (100.0)	0.063
	>9 years	4 (66.7)	5 (45.5)	2 (40.0)	2 (22.2)	0 (0.0)	
Sex	Male	3 (50.0)	7 (63.6)	5 (100.0)	7 (77.8)	0 (0.0)	0.469
	Female	3 (50.0)	4 (36.4)	0 (0.0)	2 (22.2)	1 (100.0)	
Histological Type	Classic	5 (83.3)	6 (54.5)	3 (60.0)	7 (77.8)	0 (0.0)	
	Desmoplastic/Nodular	0 (0.0)	5 (45.5)	1 (20.0)	1 (11.1)	0 (0.0)	0.358
	Extensive Nodularity	0 (0.0)	0 (0.0)	1 (20.0)	0 (0.0)	0 (0.0)	
	Large Cell/Anaplastic	1 (16.7)	0 (0.0)	0 (0.0)	1 (11.1)	1 (100.0)	
p53 Status	Wild-type	6 (100.0)	0 (0.0)	4 (80.0)	5 (55.6)	0 (0.0)	0.681
	Mutant	0 (0.0)	11 (100.0)	1 (20.0)	4 (44.4)	1 (100.0)	
Ki-67 Index	<10%	5 (83.3)	4 (36.4)	2 (40.0)	2 (22.2)	0 (0.0)	0.141
	≥10%	1 (16.7)	7 (63.6)	3 (60.0)	7 (77.8)	1 (100.0)	
Amplification	None	6 (100.0)	6 (54.5)	5 (100.0)	6 (66.7)	0 (0.0)	
	c-MYC	0 (0.0)	2 (18.2)	0 (0.0)	1 (11.1)	0 (0.0)	0.211
	n-MYC	0 (0.0)	3 (27.3)	0 (0.0)	2 (22.2)	1 (100.0)	
Tumor Location	Vermis	3 (50.0)	5 (45.5)	1 (20.0)	3 (33.3)	0 (0.0)	
	Cerebellar hemisphere	3 (50.0)	3 (27.3)	0 (0.0)	3 (33.3)	1 (100.0)	
	Pons	0 (0.0)	2 (18.2)	2 (40.0)	0 (0.0)	0 (0.0)	0.167
	Cerebellar peduncle	0 (0.0)	0 (0.0)	2 (40.0)	3 (33.3)	0 (0.0)	
	Cerebellopontine angle cistern	0 (0.0)	1 (9.1)	0 (0.0)	0 (0.0)	0 (0.0)	
Tumor Size	<3 cm	2 (33.3)	2 (18.2)	1 (20.0)	0 (0.0)	0 (0.0)	
	3–6 cm	3 (50.0)	8 (72.7)	4 (80.0)	5 (55.6)	1 (100.0)	
	>6 cm	1 (16.7)	1 (9.1)	0 (0.0)	4 (44.4)	0 (0.0)	0.060
Genetic Syndrome	None	6 (100.0)	10 (90.9)	5 (100.0)	8 (88.9)	1 (100.0)	
	Li-Fraumeni syndrome	0 (0.0)	1 (9.1)	0 (0.0)	0 (0.0)	0 (0.0)	
	Neurofibromatosis	0 (0.0)	0 (0.0)	0 (0.0)	1 (11.1)	0 (0.0)	0.409
pT Stage	pT1	1 (16.7)	1 (9.1)	0 (0.0)	0 (0.0)	0 (0.0)	
	pT2	3 (50.0)	5 (45.5)	1 (20.0)	2 (22.2)	1 (100.0)	
	pT3a–3b	1 (16.7)	5 (45.5)	3 (60.0)	5 (55.6)	0 (0.0)	0.104
	pT4	1 (16.7)	0 (0.0)	1 (20.0)	2 (22.2)	0 (0.0)	
Metastasis	Absent	4 (66.7)	5 (45.5)	3 (60.0)	1 (11.1)	0 (0.0)	
	Present	2 (33.3)	6 (54.5)	2 (40.0)	8 (88.9)	1 (100.0)	0.153
Surgical Status	GTR	5 (83.3)	7 (63.6)	1 (20.0)	2 (22.2)	0 (0.0)	
	NTR	0 (0.0)	1 (9.1)	1 (20.0)	2 (22.2)	0 (0.0)	0.064
	STR	1 (16.7)	3 (27.3)	3 (60.0)	5 (55.6)	1 (100.0)	
Radiotherapy	No	0 (0.0)	2 (18.2)	2 (40.0)	3 (33.3)	1 (100.0)	
	Yes	6 (100.0)	9 (81.8)	3 (60.0)	6 (66.7)	0 (0.0)	0.191
Chemotherapy	No	0 (0.0)	3 (27.3)	3 (60.0)	3 (33.3)	1 (100.0)	
	Yes	6 (100.0)	8 (72.7)	2 (40.0)	6 (66.7)	0 (0.0)	0.064

Footnote: MB, medulloblastoma; WNT, wingless/integrated; SHH, sonic hedgehog; p53, tumor protein 53; Ki-67, Ki-67 proliferation index; c-MYC, cellular myelocytomatosis oncogene; n-MYC, neuroblastoma MYC oncogene; pT, pathological tumor stage; GTR, gross total resection; NTR, near-total resection; STR, subtotal resection.

**Table 2:** Relationship between B7-H3 and CD47 expression and clinicopathological characteristics in medulloblastoma.

Variable	Category	B7-H3 Negative/Low *n* (%)	B7-H3 High *n* (%)	*p*	CD47 Negative/Low *n* (%)	CD47 High *n* (%)	*p*
Age	≤9 years	7 (63.6)	12 (57.1)		6 (46.2)	13 (68.4)	
	>9 years	4 (36.4)	9 (42.9)	0.513	7 (53.8)	6 (31.6)	0.186
Sex	Male	7 (63.6)	15 (71.4)		7 (53.8)	15 (78.9)	
	Female	4 (36.4)	6 (28.6)	0.474	6 (46.2)	4 (21.1)	0.133
Histological Type	Classic	6 (54.5)	15 (71.4)		8 (61.5)	13 (68.4)	
	Desmoplastic/ Nodular	3 (27.3)	4 (19.0)		3 (23.1)	4 (21.1)	
	Extensive Nodularity	1 (9.1)	0 (0.0)	0.477	0 (0.0)	1 (5.3)	0.522
	Large Cell/Anaplastic	1 (9.1)	2 (9.5)		2 (15.4)	1 (5.3)	
p53 Status	Wild-type	6 (54.5)	9 (42.9)		6 (46.2)	9 (47.4)	
	Mutant	5 (45.5)	12 (57.1)	0.398	7 (53.8)	10 (52.6)	0.615
Ki-67 Index	<10%	6 (54.5)	7 (33.3)		8 (61.5)	6 (31.6)	
	≥10%	5 (45.5)	14 (66.7)	0.556	5 (38.5)	13 (68.4)	0.094
Amplification	None	10 (90.9)	13 (61.9)		10 (76.9)	13 (68.4)	
	c-MYC	0 (0.0)	3 (14.3)		1 (7.7)	2 (10.5)	
	n-MYC	1 (9.1)	5 (23.8)	0.143	2 (15.4)	4 (21.1)	0.624
Tumor Location	Vermis	3 (27.3)	9 (42.9)		4 (30.8)	8 (42.1)	
	Cerebellar hemisphere	3 (27.3)	7 (33.3)		5 (38.5)	5 (26.3)	
	Pons	1 (9.1)	3 (14.3)		2 (15.4)	2 (10.5)	
	Cerebellar peduncle	4 (36.4)	1 (4.8)		2 (15.4)	3 (15.8)	
	Cerebellopontine angle cistern	0 (0.0)	1 (4.8)	0.182	0 (0.0)	1 (5.3)	0.992
Tumor Size	<3 cm	1 (9.1)	4 (19.0)		3 (23.1)	2 (10.5)	
	3–6 cm	9 (81.8)	12 (57.1)		7 (53.8)	14 (73.7)	
	>6 cm	1 (9.1)	5 (23.8)	0.830	3 (23.1)	3 (15.8)	0.806
Genetic Syndrome	None	11 (100.0)	19 (90.5)		11 (84.6)	19 (100.0)	
	Li-Fraumeni syndrome	0 (0.0)	1 (4.8)		1 (7.7)	0 (0.0)	
	Neurofibromatosis	0 (0.0)	1 (4.8)	0.325	1 (7.7)	0 (0.0)	0.100
pT Stage	pT1	0 (0.0)	2 (9.5)		1 (7.7)	1 (5.3)	
	pT2	6 (54.5)	6 (28.6)		3 (23.1)	9 (47.4)	
	pT3a–3b	4 (36.4)	10 (47.6)		8 (61.5)	6 (31.6)	
	pT4	1 (9.1)	3 (14.3)	0.681	1 (7.7)	3 (15.8)	0.691
Metastasis	Absent	5 (45.5)	9 (42.9)		4 (30.8)	10 (52.6)	
	Present	6 (54.5)	12 (57.1)	0.590	9 (69.2)	9 (47.4)	0.195
Surgical Status	GTR	6 (54.5)	10 (47.6)		4 (30.8)	12 (63.2)	
	NTR	0 (0.0)	4 (19.0)		1 (7.7)	3 (15.8)	
	STR	5 (45.5)	7 (33.3)	0.882	8 (61.5)	4 (21.1)	0.032
Radiotherapy	No	0 (0.0)	7 (33.3)		4 (30.8)	3 (15.8)	
	Yes	11 (100.0)	14 (66.7)	0.025	9 (69.2)	16 (84.2)	0.281
Chemotherapy	No	1 (9.1)	9 (42.9)		4 (30.8)	6 (31.6)	
	Yes	10 (90.9)	12 (57.1)	0.056	9 (69.2)	13 (68.4)	0.636

Footnote: B7-H3, B7 homolog 3; CD47, cluster of differentiation 47; Ki-67, Ki-67 proliferation index; c-MYC, cellular myelocytomatosis oncogene; n-MYC, neuroblastoma MYC oncogene; pT, pathological tumor stage; GTR, gross total resection; NTR, near-total resection; STR, subtotal resection.

**Table 3:** Association of B7-H3 and CD47 Expression with Molecular Subtypes in Medulloblastoma.

Variable	Category	B7-H3 Negative/Low *n* (%)	B7-H3 High *n* (%)	*p*	CD47 Negative/Low *n* (%)	CD47 High *n* (%)	*p*
Molecular Subtype	MB-WNT	3 (27.3)	3 (14.3)		2 (15.4)	4 (21.1)	
	MB-SHH p53 mutant	3 (27.3)	8 (38.1)		5 (38.5)	6 (31.6)	
	MB-SHH p53 wild-type	3 (27.3)	2 (9.5)		2 (15.4)	3 (15.8)	
	MB-Group 3	2 (18.2)	7 (33.3)		4 (30.8)	5 (26.3)	
	MB-Group 4	0 (0.0)	1 (4.8)	0.366	0 (0.0)	1 (5.3)	0.970

Footnote: B7-H3, B7 homolog 3; CD47, cluster of differentiation 47; MB, medulloblastoma; WNT, wingless/integrated; SHH, sonic hedgehog.

**Table 4:** Univariate Cox regression analysis for overall survival and disease-free survival in medulloblastoma.

Variable (OS)	HR	95% CI	*p*	Variable (DFS)	HR	95% CI	*p*
Age	1.803	0.694–4.689	0.226	Age	1.874	0.732–4.801	0.190
Sex	1.578	0.581–4.285	0.370	Sex	0.794	0.258–2.448	0.688
Histologic subtype	1.024	0.617–1.698	0.928	Histologic subtype	1.035	0.593–1.806	0.903
Molecular subtype	1.014	0.675–1.524	0.947	Molecular subtype	0.786	0.512–1.207	0.272
TP53 status	1.698	0.640–4.507	0.288	TP53 status	0.879	0.317–2.437	0.805
Ki-67 index	1.320	0.495–3.519	0.578	Ki-67 index	1.420	0.552–3.653	0.467
MYC amplification	0.913	0.491–1.701	0.775	MYC amplification	0.973	0.544–1.738	0.925
Tumor location	0.881	0.591–1.314	0.536	Tumor location	0.856	0.583–1.257	0.428
Tumor size	1.760	0.738–4.196	0.202	Tumor size	1.008	0.413–2.464	0.986
Genetic predisposition	1.869	1.112–3.142	0.018	Genetic predisposition	1.994	0.776–5.122	0.152
pT stage	1.420	0.780–2.585	0.251	pT stage	0.719	0.349–1.478	0.369
Metastasis	1.973	0.727–5.357	0.182	Metastasis	0.546	0.189–1.575	0.263
Surgical extent	1.258	0.764–2.073	0.367	Surgical extent	0.844	0.499–1.428	0.527
Radiotherapy	0.087	0.028–0.270	<0.001	Radiotherapy	0.184	0.046–0.736	0.017
Chemotherapy	0.266	0.101–0.704	0.008	Chemotherapy	0.495	0.170–1.440	0.197
B7-H3 expression	25.791	3.158–210.655	0.002	B7-H3 expression	136.231	1.116–16,625.955	0.045
CD47 expression	4.816	1.061–21.863	0.042	CD47 expression	1.959	0.688–5.574	0.208

Footnote: HR, hazard ratio; CI, confidence interval; OS, overall survival; DFS, disease-free survival; TP53, tumor protein 53.

**Table 5:** Multivariate Cox regression analysis for overall survival and disease-free survival in medulloblastoma.

Variable (OS)	HR	95% CI	*p*	Variable (DFS)	HR	95% CI	*p*
Genetic predisposition	1.230	0.723–2.091	0.445	–	–	–	–
Radiotherapy	0.252	0.022–2.845	0.265	Radiotherapy	0.197	0.048–0.804	0.024
Chemotherapy	1.151	0.129–10.255	0.899	–	–	–	–
B7-H3 expression	31.014	3.081–312.238	0.004	B7-H3 expression	4.118	0.711–23.842	0.114
CD47 expression	0.382	0.107–1.370	0.140	–	–	–	–

Footnote: HR, hazard ratio; CI, confidence interval; OS, overall survival; DFS, disease-free survival.

## Data Availability

The data presented in this study are available on request from the corresponding author due to privacy restrictions.

## Abbreviations

The following abbreviations are used in this manuscript:

B7-H3	B7 homolog 3
CD47	Cluster of Differentiation 47
CD276	Cluster of Differentiation 276
CNS	Central Nervous System
DFS	Disease-Free Survival
OS	Overall Survival
IHC	Immunohistochemistry
IRS	Immunoreactivity Score
HR	Hazard Ratio
CI	Confidence Interval
MB	Medulloblastoma
WNT	Wingless/Integrated
SHH	Sonic Hedgehog
GTR	Gross Total Resection
NTR	Near-Total Resection
STR	Subtotal Resection
ECOG PS	Eastern Cooperative Oncology Group Performance Status
RECIST	Response Evaluation Criteria in Solid Tumors
NCCN	National Comprehensive Cancer Network
WHO	World Health Organization
H&E	Hematoxylin and Eosin
VIF	Variance Inflation Factor
